# Association between Community Ambulation Walking Patterns and Cognitive Function in Patients with Parkinson's Disease: Further Insights into Motor-Cognitive Links

**DOI:** 10.1155/2015/547065

**Published:** 2015-10-29

**Authors:** Aner Weiss, Talia Herman, Nir Giladi, Jeffrey M. Hausdorff

**Affiliations:** ^1^Center for the Study of Movement, Cognition, and Mobility, Department of Neurology, Tel Aviv Sourasky Medical Center, 64239 Tel Aviv, Israel; ^2^Department of Neurology, Sackler Faculty of Medicine, Tel Aviv University, 6997801 Tel Aviv, Israel; ^3^Sagol School of Neuroscience, Tel Aviv University, 6997801 Tel Aviv, Israel; ^4^Sieratzki Chair of Neurology, Tel Aviv University, 6997801 Tel Aviv, Israel; ^5^Department of Physical Therapy, Sackler Faculty of Medicine, Tel Aviv University, 6997801 Tel Aviv, Israel

## Abstract

*Background*. Cognitive function is generally evaluated based on testing in the clinic, but this may not always reflect real-life function. We tested whether parameters derived from long-term, continuous monitoring of gait are associated with cognitive function in patients with Parkinson's disease (PD). *Methods*. 107 patients with PD (age: 64.9 ± 9.3 yrs; UPDRS motor sum “off”: 40.4 ± 13.2; 25.23% women) wore a 3D accelerometer on their lower back for 3 days. Computerized measures of global cognitive function, executive function, attention, and nonverbal memory were assessed. Three-day acceleration derived measures included cadence, variability, bilateral coordination, and dynamic postural control. Associations between the acceleration derived measures and cognitive function were determined. *Results*. Linear regression showed associations between vertical gait variability and cadence and between global cognitive score, attention, and executive function (*p* ≤ 0.048). Dynamic postural control was associated with global cognitive score and attention (*p* ≤ 0.027). Nonverbal memory was not associated with the acceleration-derived measures. *Conclusions*. These findings suggest that metrics derived from a 3-day worn body-fixed sensor reflect cognitive function, further supporting the idea that the gait pattern may be altered as cognition declines and that gait provides a window into cognitive function in patients with PD.

## 1. Introduction

Patients with Parkinson's disease (PD) suffer from both motor [1–4] and nonmotor disturbances [5–9]. Nonmotor deficits include cognitive changes, most notably changes in executive function and attention [6, 10–28]. Motor symptoms in PD, in particular, alterations in gait, have been associated with these cognitive deficits [29–33]. For example, cognitive impairment is related to disease severity, more specifically to bradykinesia, rigidity, and more symmetric distribution of the motor symptoms [30], as well as to axial symptoms [30, 33]. Cognitive deficits have also been related to poorer functional performance and fine motor tasks [29]. Moreover, cognitive deterioration has been linked with dual task walking abilities [34–36], freezing of gait [11, 37–40], and falls [41–46]. Imaging studies also support the link between gait and cognitive function in patients with PD [47–50]. In addition, we and others have shown that the enhancement of cognitive function may ameliorate mobility in patients with PD, that is, improve specific aspects such as time to complete certain motor tasks, increase turning speed [51], and reduce fall risk [52]. It has also been shown that methylphenidate, a catecholaminergic reuptake inhibitor, may improve both gait and sustained attention in PD [53–55]. Moreover, interventions that demand focused attention to the quality of movement such as treadmill training in a virtual reality environment [56], Tai Chi [57], and tango dancing [58, 59] may also improve gait and cognitive function and reduce the risk of falls [56–59]. Together, these cross-sectional and intervention studies suggest that there are strong links between gait and cognition in patients with PD.

Previous investigations that described these cognitive-motor associations were generally performed in laboratory or clinical settings. However, testing in these conditions may not fully reflect the interactions between gait and cognition since assessments at a single time point may be affected by motor response fluctuations, medication effects, white coat and reverse white coat behavior, and the Hawthorne effect (also known as the observer effect; e.g., individuals modify their behavior while being observed). Studies are needed to evaluate the cognitive-gait relationship in real-life environments and to assess how cognition is associated not just with what the subject “can do,” in sterile laboratory conditions, but with what the subject “does do” in everyday life. To address this question, we tested whether measures derived from long-term, continuous gait monitoring are associated with cognitive function in patients with PD. Based on the previously described relationships between gait and executive function and attention in PD and in other groups, in this exploratory investigations, we focused on these associations. We also examined nonverbal memory, putatively a cognitive measure that would not be related to everyday walking abilities.

## 2. Methods

### 2.1. Participants

110 patients with PD participated in a cross-sectional study focusing on PD motor subtypes [60]. They were recruited from the outpatient Movement Disorders Unit at the Tel Aviv Medical Center and from other affiliated clinics. Three subjects were excluded due to technical problems (device failure or loss). Thus, data from 107 patients was analyzed in the present study. Subjects were included if they were diagnosed by movement disorders specialist with idiopathic PD (as defined by the UK Brain Bank criteria), were between 40 and 85 years of age, had a Hoehn and Yahr score between I and IV, were ambulatory, and had a Mini Mental State Examination (MMSE) score above 24 points. Subjects were nondemented; however, one cannot rule out the possibility that mild cognitive impairment (MCI) was present in some of the subjects. Subjects were excluded if they have had brain surgery or had significant comorbidities likely to affect gait, for example, acute illness, orthopedic disease, or history of stroke. Subjects who could not walk in the off medication cycle and subjects who could not comply with the protocol were excluded. Ethics approval from the human studies committee of the Tel Aviv Sourasky Medical Center was obtained and all participants provided informed written consent, according to the Declaration of Helsinki.

### 2.2. Assessment of PD Symptoms and Cognitive Function

Parkinsonian symptoms, disease duration, and disease severity were assessed based on an interview and the Unified Parkinson's Disease Rating Scale (MDS-UPDRS) [61], both “on” and “off” medications. A previously validated, computerized neuropsychological test battery (NeuroTrax; Modiin, Israel) quantified specific aspects of cognition during the on medication phase. The cognitive measures that were assessed included aspects of executive function, memory (specifically, nonverbal memory), and attention. The executive function index score was based on the Go-No-Go (composite score), Stroop interference (composite score), and catch game (total score of a hand-eye coordination task) tests. The memory index score included the nonverbal memory (total accuracy) and delayed nonverbal memory (accuracy) tests. The attention index was based on the Go-No-Go (response time and response time standard deviation) and Stroop interference (response time) tests. Scores for these cognitive indices were on an IQ-like scale, with 100 representing the average score in cognitively intact individuals, normalized for age and education. A global cognitive score based on the average across the memory, executive function, attention, and motor skills measures was also determined using this battery [62–64]. These tests were previously used to assess cognitive function in patients with PD in cross-sectional studies [16, 36, 53, 65–67] and have also been responsive to interventions in PD [51, 53].

### 2.3. Three-Day Assessment of Gait and Mobility

After undergoing the clinical assessment, patients wore a sensor on their lower back for 3 consecutive days while taking their normal medications. The data acquisition device and signal processing were described previously [68–71]. Briefly, participants wore a small, lightweight sensor (McRoberts, DynaPort Hybrid system, Netherlands) on a belt on the lower back. The units' dimensions are 87 × 45 × 14 mm (74 grams). The Hybrid includes a triaxial accelerometer (sensor range and resolution are ±2 g and ±1 mg, resp.) and a triaxial gyroscope (data not analyzed in the present study). The 3 acceleration axes studied were the vertical, mediolateral, and anterior-posterior. Data was saved on an SD card at 100 Hz and later transferred to a personal computer for further analysis (using Matlab, the Mathworks software).

The data analysis of the 3-day recordings included two stages [69–71]: (1) detection of walking segments above one minute and (2) application of acceleration derived measures to the walking segments that were identified in the previous stage. Walking segments of above one minute were chosen to ensure that the intervals represented consistent walking. Metrics that reflect the quantity and quality of the walking activity were determined in the second stage. Quantity measures included percent of overall time spent walking, the total number of steps, and median cadence per bout. Quality related sensor-derived measures included amplitude of the dominant frequency in the power spectral density domain, which is a frequency-derived measure that reflects variability of the gait pattern [68–71], stride regularity which reflects gait rhythmicity and consistency [72], and the harmonic ratio which is an index of gait smoothness [73]. We also derived the phase coordination index [74] which is a measure of bilateral, left-right coordination and reflects the consistency and accuracy of phase generation. A lower phase coordination index reflects a more consistent and accurate phase generation.

### 2.4. Statistical Analyses

Statistical analyses were performed using SPSS version 21. We examined whether the different cognitive measures (i.e., executive function, memory, attention, and global cognitive score) were associated with the 3-day acceleration measures. This was done using univariate and multivariate linear regression models, with each cognitive measure as the dependent variable. In the first stage, we applied a univariate model with the Enter method for each acceleration measure separately. In the next stage, we chose all the acceleration measures that were significant in the first stage, and after making sure that they were not significantly correlated with each other (Spearman *r* < 0.8), we inserted them in a multivariate model. We applied the multivariate model with the stepwise method to determine which measures remain significant. Both univariate and multivariate models were adjusted for age, gender, and disease duration. In addition, to further explore the gait-cognitive associations, for each cognitive measure, we compared the lowest (i.e., worst) quartile (*N* = 26) to the highest (i.e., best scores) quartile (*N* = 27). Normality was assessed using the Kolmogorov-Smirnov test. Based on this check, either Student's *t*-tests or the Mann-Whitney test was used to compare the participants with low and high cognitive function. Corrections for multiple comparisons were made using the Hochberg-Benjamini method.

## 3. Results

### 3.1. Subject Characteristics

Data from a cohort of 107 patients with PD (age: 64.9 ± 9.3 yrs; UPDRS motor score “off”: 40.4 ± 13.2; Hoehn and Yahr “off”: 2.6 ± 0.7; 25.2% women) was analyzed. In addition, from this cohort, the lowest and highest cognitive measure quartiles were derived.

### 3.2. Global Cognitive Score

In the entire cohort (see Table 1(a)), global cognitive score was associated with cadence (*p* = 0.003), vertical stride regularity (*p* = 0.015), vertical amplitude (*p* = 0.009), anterior-posterior harmonic ratio (*p* = 0.038) and dynamic postural control, as reflected by the mediolateral amplitude (*p* = 0.027). In the adjusted multivariate model, global cognitive score was associated with cadence (*p* = 0.013) and vertical stride regularity (*p* = 0.048). When comparing the lowest and highest global cognitive score quartiles (see Table 1(b)), the mean global cognitive score of the low global cognitive score quartile was 77.72 ± 10.44 and 106.74 ± 4.01 for the high global cognitive score quartile. Age, gender, and disease duration did not differ (*p* ≥ 0.252) between subjects in the low and high global cognitive score quartiles. Quantity of walking over the 3 days was similar in the high and low global cognitive score quartiles (*p* ≥ 0.076). In contrast, measures related to the quality of gait in the vertical acceleration axis (V) differed in the two groups. People with higher global cognitive score had higher (better) gait consistency, as expressed by the higher V stride regularity (*p* = 0.008), and lower (better) step variability, as expressed by the higher V amplitude (*p* = 0.019), as well as higher (better) gait smoothness, as expressed by the higher V harmonic ratio (*p* = 0.039). Bilateral coordination, as reflected by the phase coordination index, and dynamic postural control, as reflected by the mediolateral amplitude, stride regularity, and harmonic ratio measures, were not significantly different in subjects with low and high global cognitive scores (*p* ≥ 0.075).

### 3.3. Executive Function

In the entire cohort (see Table 2(a)), executive function was associated with cadence (*p* = 0.013) and vertical acceleration amplitude (*p* = 0.016). In the adjusted multivariate model, executive function was associated with the vertical acceleration amplitude (*p* = 0.009). When comparing the lowest and highest executive function quartiles (see Table 2(b)), the mean executive function score of the low executive function quartile was 76.85 ± 10.81 and 109.59 ± 4.57 for the high executive function quartile. Age, gender, and disease duration did not differ (*p* ≥ 0.132) between subjects in the low and high executive function score quartiles. Quantity of walking over the 3 days was similar in the high and low executive function quartiles, with the exception of cadence, which was lower in the group with low executive function (*p* = 0.009). People with higher executive function had better bilateral coordination, that is, a lower (“better”) phase coordination index (*p* = 0.033). People with high executive function also tended to have a lower step variability and higher gait smoothness, as expressed by the higher vertical acceleration amplitude (*p* = 0.035) and higher anterior-posterior harmonic ratio (*p* = 0.038) (however, these differences were not statistically significant after correcting for multiple comparisons). Dynamic postural control, as reflected by the mediolateral amplitude, stride regularity, and harmonic ratio measures, was not significantly different between groups (*p* ≥ 0.254).

### 3.4. Attention

In the entire cohort (see Table 3(a)), attention was associated with cadence (*p* < 0.001), vertical acceleration amplitude (*p* = 0.001), vertical stride regularity (*p* = 0.007), anterior-posterior harmonic ratio (*p* = 0.025), and dynamic postural control as reflected by the mediolateral amplitude (*p* = 0.026). In the adjusted multivariate model, attention was associated with cadence (*p* = 0.0001) and with the vertical amplitude (*p* = 0.017). When evaluating the lowest and highest attention quartiles (see Table 3(b)), the mean attention score of the low attention quartile was 70.63 ± 17.94 and 108.41 ± 3.53 for the high attention quartile. Age, gender, and disease duration did not differ (*p* ≥ 0.694) between subjects in the low and high attention quartiles. Quantity of walking over the 3 days was similar in the high and low attention quartiles, with the exception of cadence, which was lower in the group with lower attention (*p* = 0.009). For the quality of gait measures, participants with high attention had lower step variability, as expressed by the higher vertical amplitude (*p* = 0.013). Bilateral coordination, as reflected by the phase coordination index, was not significantly different between the groups (*p* = 0.278). The groups also had similar results in dynamic postural control, as reflected by the mediolateral amplitude, stride regularity, and harmonic ratio measures (*p* ≥ 0.393).

### 3.5. Nonverbal Memory

In the entire cohort, none of the acceleration measures were associated with nonverbal memory. When evaluating the lowest and highest memory quartiles, the mean memory score of the low memory quartile was 76.34 ± 10.02 and 112.73 ± 4.39 for the high memory quartile. Age, gender, and disease duration did not differ (*p* ≥ 0.344) between subjects in the low and high memory quartiles. None of the gait quantity or quality measures were different between the groups in the low and high memory quartiles (*p* ≥ 0.112).

## 4. Discussion

Previous studies demonstrated an association between poorer executive function and attention and between gait dysfunction in patients with PD [11, 36, 37, 75–77]. Many daily living activities normally involve complex, sometimes multiple, actions and may therefore be compared with dual task activities in the lab, which mainly rely on executive function and the ability to divide attention [35, 78]. For example, previous in-lab studies demonstrated that gait speed and stride length become worse under dual task conditions in patients with PD [34, 43, 46, 79]. Consistent with our hypotheses, here we show, for the first time, as far as we know, that lower executive function, attention, and global cognitive score are associated with gait changes quantified during everyday walking, especially those that reflect the consistency and step-to-step variability of the walking pattern.

Earlier in-lab studies found that stride-to-stride variability becomes worse in response to dual task conditions in patients with PD [34, 36, 80]. Here, we observed that increased gait variability is associated with lower executive function, attention, and global cognitive score, consistent with those in-lab findings. Previously, gait asymmetry was shown to worsen in response to dual task walking [66, 81]. Here, we show that bilateral coordination, which is indirectly related to gait asymmetry, is worse in patients with lower executive function (although bilateral coordination was not significantly related to attention). While the above studies assessed short gait intervals in laboratory and clinical tests, here, we demonstrate the relationship between relatively long-term gait assessment and cognition.

The present findings demonstrate that an association exists between certain gait and motor aspects in the participant's natural setting and between particular cognitive measures. The results suggest that distinct elements of cognition differ in their association with gait properties. While executive function, attention, and global cognitive function were related to some gait features, memory was not. This is consistent with previous studies [16, 36], which showed that executive function and attention, but not memory, were impaired in patients with PD compared to controls and that only executive function was related to gait while dual tasking in patients with PD [36]. It is interesting to observe that these different effects were also seen when examining gait in daily life, outside of the laboratory.

Compared to the motor assessment in the lab or clinic, daily living assessment using body-fixed sensors allows for a long-term, real-life quantitative method for obtaining information regarding the patient's symptoms. We speculate that ambulatory quantitative assessment will allow users, clinicians, and scientists to obtain more relevant data, in addition to the clinical evaluation [82]. These initial findings suggest that metrics derived from a 3-day worn body-fixed sensor are sensitive to cognitive changes in PD patients as they walk in their routine daily living environment, further supporting the idea that the gait pattern may be altered as cognition declines and that gait can provide a window into cognition. Cognitive demands of walking in complex and challenging daily living environment may be the source for this link. Interestingly, previous studies suggest that cholinergic deficits in PD may be the common basis for certain aspects of both cognitive and gait disturbances [13, 47, 83–91]. In the future, it may be interesting to investigate its role in the observed associations.

This study has several limitations. For example, this work was cross-sectional and does not provide evidence for cause and effect. Both gait and cognition often fluctuate throughout the day and may be influenced by dopaminergic, anticholinergic, and antifreezing medications. Furthermore, the MCI status of the participants was not explicitly evaluated; this may have acted as a confounder. Another issue to be considered is that the cognitive measures used in this study were not as comprehensive as a more detailed neuropsychological battery and thus only sampled a subset of cognitive functions. For example, we evaluated only nonverbal memory but did not assess verbal memory. Nonverbal (e.g., abstract figures and faces) memory decline has been shown to be more typical in right hemisphere disease, while verbal memory (names and sentences) has been shown to be more related to left hemisphere lesions [92]. While both of these memory types seem to decline in PD, greater decline is generally seen in verbal rather than nonverbal memory [92]. One advantage of using nonverbal memory is that it is largely independent of language skills. It would, therefore, be important to quantify both types of memory in future work. Similarly, executive function refers to a wide array of cognitive functions, while in the present study we focused primarily on response inhibition and switching aspects of executive function. In the future, it would be informative to assess the relationship to other aspects of executive function and perhaps also to investigate the relationship between the motor measures and complex tasks such as instrumental activities of daily living (IADL). Nonetheless, while future work promises to be informative, the cognitive battery employed here does provide initial insight. It is based on standardized tests that have been widely used to assess cognitive function in PD and other cohorts [34, 51, 81, 93–99]. Thus, while not completely comprehensive, the assessment of cognition used in this study provides initial insight into the association between cognition and everyday gait in PD.

Future studies should further investigate the relationship between cognitive decline and various continuous and episodic gait disturbances in the home setting, as well as motor fluctuations and response to medications at different times of the day. Study of visuocognitive tasks in relation to daily living gait may also help to further delineate the important role of vision in gait. Assessment of shorter gait bouts may also provide additional information regarding quantitative performance features and its relationship to cognitive function. This was an initial study. In the future it may be interesting to evaluate whether changes in gait as measured in the home setting are sensitive to short-term changes in the cognitive state, for example, due to drug effects, side effects, or anticholinesterase efficacy, to investigate additional gait and cognitive measures, to perform more comprehensive neurophysiological assessments, and to examine how the relationships observed change in a prospective study. The results of this exploratory investigation set the stage for those future studies.

## Figures and Tables

**Table:** (a)

	Adjusted univariate model	Adjusted multivariate model
Quantity measures (activity count)		
Total percent of activity duration [%]		
AP	−0.01 (−1.32–1.14) *p* = 0.88	—
Total number of steps for 3 days [#]		
AP	0.03 (−0.0002–0.0002) *p* = 0.77	—
Cadence [steps/minute]		
AP	**0.29 (0.09–0.45)** **p** = 0.003	**0.24 (0.04–0.39)** **p** = 0.013

Quality of activity measures		
Phase coordination index [%]		
AP	−0.08 (−0.40–0.16) *p* = 0.390	—
Amplitude of dominant frequency [prs]		
V	**2.65 (4.18–28.91)** **p** = 0.009	—
AP	1.59 (−3.35–28.80) *p* = 0.11	—
ML	**−0.23 (−34.83–−2.10) ** **p** = 0.027	—
Stride regularity [g^2^]		
V	**0.24 (4.09–37.75)** **p** = 0.015	**0.18 (0.14–2.37)** **p** = 0.048
AP	0.16 (−3.90–41.70) *p* = 0.10	—
ML	−0.28 (−22.00–16.73) *p* = 0.78	—
Harmonic ratio		
V	0.15 (−0.91–8.00) *p* = 0.11	—
AP	**0.20 (0.27–9.67)** **p** = 0.038	—
ML	0.08 (−9.55–2290) *p* = 0.41	—

^*∗*^Entries are the *B* values, 95% confidence intervals, and the associated *p* value. Univariate and multivariate models were adjusted for age, gender, and disease duration.

**Table:** (b)

Measure	PD low global cognitive score	PD high global cognitive score	*p* value
Quantity measures (activity count)			
Total percent of activity duration [%]			
AP	2.39 ± 2.24	2.21 ± 2.13	0.618^†^
Total number of steps for 3 days [#]			
AP	10346.38 ± 9533.69	10063.80 ± 10791.24	0.618^†^
Cadence [steps/minute]			
AP	101.46 ± 17.73	108.92 ± 11.76	0.076

Quality of activity measures			
Phase coordination index [%]			
AP	7.77 ± 8.89	6.51 ± 7.43	0.075^†^
Amplitude of dominant frequency [prs]			
V	**0.56 ± 0.19**	**0.69 ± 0.16**	**0.019** ^*∗*^
AP	0.56 ± 0.15	0.61 ± 0.16	0.245
ML	0.19 ± 0.15	0.17 ± 0.11	0.748^†^
Stride regularity [g^2^]			
V	**0.46 ± 0.11**	**0.56 ± 0.13**	**0.008** ^**∗**^
AP	0.50 ± 0.11	0.54 ± 0.10	0.148
ML	0.35 ± 0.14	0.36 ± 0.11	0.753
Harmonic ratio			
V	**1.98 ± 0.46**	**2.26 ± 0.48**	**0.039** ^**∗**^
AP	1.86 ± 0.43	2.09 ± 0.46	0.076
ML	0.62 ± 0.16	0.62 ± 0.10	0.902

^†^Measures which were not distributed normally according to the Kolmogorov-Smirnov test and therefore were analyzed with the Mann-Whitney test.

^*∗*^Measures which were significantly different in the two groups. We performed the Hochberg-Benjamini method for multiple comparison analysis for each of the 3 locomotor constructs separately: vertical (V), anterior-posterior (AP), and mediolateral (ML). *p* values less than or equal to 0.039 (V), 0.05 (AP), and 0.05 (ML) were considered statistically significant in the 3 different constructs in the present analyses.

**(a):** 

	Adjusted univariate model	Adjusted multivariate model
Quantity measures (activity count)		
Total percent of activity duration [%]		
AP	−0.02 (−1.53–1.18) *p* = 0.80	—
Total number of steps for 3 days [#]		
AP	0.01 (−0.0002–0.0003) *p* = 0.87	—
Cadence [steps/minute]		
AP	**0.25 (0.05**–**0.45) ** **p** = 0.013	—

Quality of activity measures		
Phase coordination index [%]		
AP	−0.09 (−0.45–0.16) *p* = 0.357	—
Amplitude of dominant frequency [prs]		
V	**0.24 (3.24**–**30.23) ** **p** = 0.016	**0.25 (4.43**–**0.54) ** **p** = 0.009
AP	0.16 (−2.70–32.64) *p* = 0.096	—
ML	−0.15 (−31.67–4.85) *p* = 0.14	—
Stride regularity [g^2^]		
V	0.16 (−3.10–34.60) *p* = 0.10	—
AP	0.13 (−8.34–42.09) *p* = 0.18	—
ML	−0.05 (−26.68–15.93) *p* = 0.61	—
Harmonic ratio		
V	0.08 (−2.97–6.95) *p* = 0.42	—
AP	0.18 (−0.13–10.25) *p* = 0.056	—
ML	0.09 (−9.43–26.28) *p* = 0.35	—

^*∗*^Entries are the *B* values, 95% confidence intervals, and the associated *p* value. Univariate and multivariate models were adjusted for age, gender, and disease duration.

**(b):** 

Measure	PD low executive function	PD high executive function	*p* value
Quantity measures (activity count)			
Total percent of activity duration [%]			
AP	2.21 ± 2.29	2.32 ± 2.11	0.866
Total number of steps for 3 days [#]			
AP	9324.44 ± 9707.57	10979.40 ± 10573.83	0.498^†^
Cadence [steps/minute]			
AP	**98.83 ± 16.02**	**108.62 ± 9.54**	**0.009** ^**∗**^

Quality of activity measures			
Phase coordination index [%]			
AP	9.66 ± 10.55	6.59 ± 7.38	0.033^†^
Amplitude of dominant frequency [prs]			
V	0.58 ± 0.21	0.70 ± 0.19	0.035
AP	0.55 ± 0.17	0.60 ± 0.14	0.243
ML	0.20 ± 0.14	0.16 ± 0.12	0.254^†^
Stride regularity [g^2^]			
V	0.49 ± 0.14	0.54 ± 0.16	0.226
AP	0.50 ± 0.11	0.53 ± 0.11	0.301
ML	0.36 ± 0.12	0.34 ± 0.11	0.600
Harmonic ratio			
V	2.12 ± 0.49	2.21 ± 0.55	0.534
AP	1.85 ± 0.41	2.09 ± 0.40	0.038
ML	0.63 ± 0.17	0.63 ± 0.09	0.863

^†^Measures which were not distributed normally according to the Kolmogorov-Smirnov test and therefore were analyzed with the Mann-Whitney test.

^*∗*^Measures which were significantly different in the two groups. We performed the Hochberg-Benjamini method for multiple comparison analysis for each of the 3 locomotor constructs separately: vertical (V), anterior-posterior (AP), and mediolateral (ML). *p* values less than or equal to 0.03 (V), 0.03 (AP), and 0.05 (ML) were considered statistically significant in the 3 different constructs in the present analyses; that is, none of the variability measures were significant.

**(a):** 

	Adjusted univariate model	Adjusted multivariate model
Quantity measures (activity count)		
Total percent of activity duration [%]		
AP	−0.03 (−2.00–1.44) *p* = 0.74	—
Total number of steps for 3 days [#]		
AP	0.02 (−0.0003–0.0004) *p* = 0.79	—
Cadence [steps/minute]		
AP	**0.45 (0.35–0.82)** **p** = 0.000003	**0.36 (0.24–0.70)** **p** = 0.0001

Quality of activity measures		
Phase coordination index [%]		
AP	−0.10 (−0.61–0.18) *p* = 0.282	—
Amplitude of dominant frequency [prs]		
V	**0.34 (12.93–46.51)** **p** = 0.001	**0.21 (3.46–34.80)** **p** = 0.017
AP	0.11 (−9.62–35.44) *p* = 0.25	—
ML	**−0.23 (−48.77–−3.18) ** **p** = 0.026	—
Stride regularity [g^2^]		
V	**0.27 (9.22–55.70) ** **p** = 0.007	—
AP	0.12 (−11.13–52.74) *p* = 0.199	—
ML	0.01 (−25.63–28.36) *p* = 0.92	—
Harmonic ratio		
V	0.19 (−0.04–12.28) *p* = 0.052	—
AP	**0.22 (0.97–14.03)** **p** = 0.025	—
ML	−0.19 (−24.88–20.50) *p* = 0.84	—

^*∗*^Entries are the *B* values, 95% confidence intervals, and the associated *p* value. Univariate and multivariate models were adjusted for age, gender, and disease duration.

**(b):** 

Measure	PD low attention	PD high attention	*p* value
Quantity measures (activity count)			
Total percent of activity duration [%]			
AP	2.27 ± 2.25	2.20 ± 2.01	0.957^†^
Total number of steps for 3 days [#]			
AP	9537.29 ± 9612.54	10187.55 ± 10479.79	0.789^†^
Cadence [steps/minute]			
AP	**99.47 ± 16.73**	**110.33 ± 11.94**	**0.009** ^**∗**^

Quality of activity measures			
Phase coordination index [%]			
AP	7.78 ± 8.97	7.53 ± 8.96	0.278^†^
Amplitude of dominant frequency [prs]			
V	**0.57 ± 0.20**	**0.70 ± 0.16**	**0.013** ^**∗**^
AP	0.57 ± 0.16	0.56 ± 0.14	0.809
ML	0.19 ± 0.15	0.16 ± 0.12	0.393
Stride regularity [g^2^]			
V	0.50 ± 0.12	0.58 ± 0.13	0.054
AP	0.53 ± 0.10	0.54 ± 0.12	0.707
ML	0.38 ± 0.14	0.38 ± 0.13	0.896
Harmonic ratio			
V	2.13 ± 0.49	2.28 ± 0.56	0.308
AP	1.96 ± 0.48	2.07 ± 0.55	0.446
ML	0.60 ± 0.16	0.63 ± 0.13	0.581

^†^Measures which were not distributed normally according to the Kolmogorov-Smirnov test and therefore were analyzed with the Mann-Whitney test.

^*∗*^Measures which were significantly different in the two groups. We performed the Hochberg-Benjamini method for multiple comparison analysis for each of the 3 locomotor constructs separately: vertical (V), anterior-posterior (AP), and mediolateral (ML). *p* values less than or equal to 0.013 (V), 0.05 (AP), and 0.05 (ML) were considered statistically significant in the 3 different constructs in the present analyses.
